# Effects of Inspiratory Muscle Training on Physiological Performance Variables in Women's Handball

**DOI:** 10.5114/jhk/169366

**Published:** 2023-09-05

**Authors:** Andrés Santiago Parodi-Feye, Álvaro Daniel Cappuccio-Díaz, Carlos Alberto Magallanes-Mira

**Affiliations:** 1 Department of Physical Education and Sport, Superior Institute of Physical Education, University of the Republic (Udelar), Montevideo, Uruguay.

**Keywords:** handball, inspiratory muscle training, aerobic capacity, POWERBreathe

## Abstract

Inspiratory muscle training (IMT) has been used in different sports, although there is no consensus on its benefits. We investigated the effects of eight weeks of IMT in women's handball. Twenty-four players were randomly distributed into experimental (EXP; n = 13) and control (CON; n = 11) groups. Only the EXP group performed IMT using the POWERBreathe device, following indications of the manufacturers. Before and after the intervention, spirometric variables were evaluated at rest and during a graded test using direct analysis of respiratory gases. Perception of exertion at submaximal intensity was also determined. No significant differences were observed post- vs. pre-intervention (p ≥ 0.05) regarding forced vital capacity (FVC), forced expiratory volume in the 1^st^ second (VEF1), FVC/VEF1, maximal expiratory flow at 50% of FVC or peak inspiratory flow. Post-intervention, only the CON group increased their absolute and relative VO_2max_ (2.1 ± 0.2 L/min pre vs. 2.2 ± 0.3 L/min post; 33.6 ± 3.6 ml/kg∙min pre vs. 34.5 ± 3.2 ml/kg∙min post, respectively). No significant improvements (p ≥ 0.05) were observed in VO_2_ associated with ventilatory threshold 1 (VT1), nor in the intensity associated with VO_2max_ and VT1. However, there was a tendency for the mentioned variables to decrease in the CON group, while in the EXP group the trend was to maintain or increase previous values. IMT did not determine an improvement in the perception of exertion at submaximal intensity. The use of POWERBreathe, as described in the present study, is feasible in terms of time and effort, although its benefits may not be significant.

## Introduction

The respiratory system of healthy young people is not usually considered a limiting factor during endurance exercise. In most subjects, no or only slight changes in O_2_ arterial partial pressure from rest to maximal effort have been observed, with only a small reduction in arterial haemoglobin saturation (Amann, 2012). However, it has been determined that ventilation's work plays a significant role in cardiovascular response during high-intensity exercise.

Wells and Norris (2009) proposed that ventilatory muscle performance decreased during efforts exceeding 85% of VO_2max_, resulting in exercise-induced arterial hypoxemia, ventilatory muscle fatigue, and dyspnea. These phenomena would be associated with a progressive increase in the recruitment of accessory inspiratory muscles with a decrease in diaphragmatic efficiency, resulting in respiratory asynchrony and an inefficient ventilatory mechanism. This would reduce diaphragmatic perfusion while increasing metabolic and blood flow requirements to these muscle groups (McConnell and Romer, 2004). Sensory stimulation of the central nervous system secondary to these events activates the metaboreflex mechanism, causing an increase in the percentage of cardiac output delivered to the inspiratory musculature. This results in up to a 20% reduction in the blood flow to the active limbs, potentially impairing performance (Harms et al., 1998; Smith et al., 2017).

Inspiratory muscles can be trained following the same principles as other skeletal muscles by employing repetitive ventilatory exercises against an external load (Elkins and Dentice, 2015). Previous studies have shown that inspiratory muscle training (IMT) significantly increases key enzymes of aerobic metabolism in the diaphragm (Akkbas et al., 1989). In addition, IMT allows attenuation of reflex vasomotor changes secondary to intense work of the ventilatory musculature, improving aerobic exercise tolerance (McConnell and Lomax, 2006).

IMT protocols have been used in different sports, with the aim of enhancing performance, as part of training programs of high-level athletes and complementary to their regular training (Cybulska and Drobnik 2015; Jurić et al., 2019; Rozek- Piechura et al., 2020). In trained swimmers, an improvement in inspiratory strength and performance in distances less than 400 m was observed after six weeks of IMT (Cirino et al., 2021; Kilding et al., 2010; Ohya et al., 2021). In competitive cycling, IMT for six weeks improved performance and decreased respiratory and the peripheral rating of perceived exertion (RPE) (Johnson et al., 2007; Romer et al., 2002). In female rowers, Volianitis et al. (2001) observed an improvement in performance after 11 weeks of IMT. A performance improvement was also observed in male rowers after four weeks of IMT, which was not verified when the expiratory musculature was trained during the same period (Griffiths and McConnell, 2007). In elite endurance runners, eight weeks of IMT increased relative VO_2max_ and decreased post-training blood lactate values (Rozek-Piechura et al., 2020).

Regarding team sports, in a study conducted on professional female soccer players, IMT for six weeks determined an attenuation of the inspiratory musculature metaboreflex when performing high-intensity exercise, resulting in increased perfusion to the extremities (Archiza et al., 2018). Also, in youth soccer players, eight weeks of IMT determined an improvement in anaerobic and interval aerobic endurance tests and exercise tolerance (Najafi et al., 2019). In male handball, Hartz et al. (2018) observed a significant increase in inspiratory muscle strength and endurance, associated with an improvement in aerobic physical performance, after 12 weeks of IMT. To the best of our knowledge, no previous studies have analyzed the effect of this type of training in women's handball.

The present study aimed to determine the effects of an IMT device called POWERBreathe Plus+ on cardiovascular and respiratory variables associated with sports performance in competitive female handball players. In addition, we sought to determine the practical feasibility of using this device in this population.

## Methods

### Participants

Thirty adult female handball players were selected through convenience sampling. All of them had medical certifications, which remained valid during the entire duration of the intervention. None of them had previously undergone IMT. Athletes were orally informed of the characteristics and objectives of the present study, after which they read and signed an informed consent form. Subsequently, they were randomly assigned to an experimental (EXP, n = 15) or a control group (CON, n = 15).

The following inclusion and exclusion criteria were considered: (i) being older than 18 years at the start of the experimental intervention; (ii) having at least two years of competitive handball experience; (iii) having answered all the questions of the PAR-Q pre-participation questionnaire negatively; (iv) not exceeding 20% of absences from handball or IMT training during the intervention period; (v) not being a smoker or having quit smoking at least six months prior to the start of the intervention; and (vi) not presenting pathologies or consuming medications that could eventually affect the results of the study.

### Design and Procedures

The present work was approved by the Ethics Committee of the University of the Republic (Udelar), Uruguay (endorsement number 3/2020). It was implemented following the principles established in the Declaration of Helsinki (Rev. 2008).

The study was conducted during the competitive period while participants continued their regular training. The training frequency was four times a week, two hours per session, during which athletes performed basic physical conditioning (approximately 60 minutes) along with technical and tactical exercises. Additionally, they had competitions on weekends. Only athletes in the EXP group underwent IMT. Before and after the intervention, all participants were evaluated, as detailed in the following section.

### Measures

The week before and after the intervention, all players were evaluated using resting spirometry and an incremental progressive test with respiratory gas analysis. They were asked not to ingest food three hours before training. In addition, they were asked not to drink or eat foods that might contain stimulant substances such as caffeine or other methylxanthines 12 hours before the test and to refrain from drinking alcohol in the last 24 hours. All the evaluation sessions were conducted in a medical clinic at an ambient temperature between 22 and 24ºC. A physician was always present during the tests.

Resting spirometry was performed following the guidelines of the American Thoracic Society and the European Respiratory Society (Gibson et al., 2002). Forced Vital Capacity (FVC), Forced Expiratory Volume in the first second (FEV1), Maximal Expiratory Flow at 50% of FVC (MEF50), and Peak Inspiratory Flow (PIF) were measured. In addition, the Tiffeneau-Pinelli index (FEV1/FVC ratio) was calculated.

The incremental progressive test was performed on a cycloergometer (Cyclus 2; RBM elektronik-automation GmbH, Leipzig, Germany). Participants were asked to pedal at a constant cadence between 60 and 80 rpm; the initial pedaling power was set at 30 W. Every 2 min, successive 30 W increments were made until the athlete reached exhaustion or could not reach the cadence necessary to maintain the requested power. The load was controlled automatically by the equipment's software.

The MetaLyzer 3B-R3 device (CORTEX Biophysik GmbH, Leipzig, Germany) was used for both tests. This equipment allows direct, breath-by-breath measurement of O_2_ and CO_2_ concentration in inspired and exhaled air, among other variables. It presents high reproducibility values, with intra-class reliability coefficients of 0.969 for VO_2_, 0.964 for VCO_2_, and 0.953 for expiratory minute volume (VE) (Meyer et al., 2001).

During the test, the participant's heart rate (HR) was also monitored employing a Polar® H7 sensor band (Polar Electro Inc., Finland). Using Bluetooth 4.0 technology, the data were received by the MetaLyzer 3B-R3 device mentioned above and interpreted by the corresponding software (MetaSoft Studio).

During the incremental progressive test, in addition to controlling the mechanical power of the cycloergometer, respiratory gases and the HR were measured continuously throughout the entire procedure. Absolute and relative VO_2max_ and their associated pulmonary ventilation (VE) as well as the respiratory exchange ratio (RER) were determined. Additionally, the first ventilatory threshold (VT1) was estimated using the v-slope methodology, obtaining its absolute and relative VO_2_, VE, RER and mechanical power. At the end of stage 4, the RPE was recorded immediately before moving on to the next stage. For this purpose, athletes were presented with a table with the modified Borg scale, and asked to indicate the RPE they perceived at that moment following its guidelines (Kilpatrick et al., 2020).

Before each assessment, athlete's body mass was measured using a GA.MA. scale (GA.MA. Italy Professional) accurate to 100 g. In addition, prior to the first evaluation, body height was determined using a SECA 213 stadiometer (SECA, Germany) with accuracy of 1 mm. Both measurements were performed under the protocols established by the International Society for the Advancement of Kinanthropometry (ISAK) (Marfell-Jones et al., 2019).

During the second evaluation, athletes in the EXP group were additionally asked about any changes in the cardiovascular and respiratory capacity they had perceived during the intervention that they could associate with IMT. They were also asked to mention how much effort it took them to use the POWERBreathe plus+ during this period. For this purpose, an analog scale from 0 to 10 was used, where 0 corresponded to “it cost me absolutely nothing, it did not represent any effort or discomfort at all”, and 10 corresponded to “it cost me a lot of time or effort to have to use this device, I find it so cumbersome that I would not use it again”.

### Inspiratory Muscle Training Protocol

The EXP group performed IMT for eight weeks, using the POWERBreathe Plus+ Heavy Resistance. This model is suggested for high-level athletes or active subjects who have reached the highest levels with the POWERBreathe Plus+ Medium Resistance. The protocol was based on the manufacturers' recommendations specified in the user manual.

On the first day, athletes were instructed about the correct use of the device. Then they all performed the first IMT session in the presence of the authors of this study, who corroborated that the technique used was adequate. From that moment on, during the first week, participants were asked to perform, from Monday to Friday and always at approximately the same time, 30 consecutive inhalations at a resistance level of 0 (minimum), which for the model used corresponds to 29 cmH_2_O. If they could not perform the 30 inhalations continuously, they were asked to briefly pause two or three “normal” respiratory cycles and then continue with IMT until completing the planned number of breaths. They were asked not to increase the device load during these first five days.

From the second week until the intervention was completed, they were asked to perform 30 breaths twice a day from Monday to Friday, in the morning and at night, and always at approximately the same time. Following the manufacturers' recommendations, if they could continuously complete the number of inspirations mentioned above, they were asked to increase the load by ¼ turn (corresponding to 28 cmH_2_O) once a day until the end of the intervention. They were also asked to note any perceived side effects of using the device (dizziness, headache, among others).

### Statistical Analysis

Data are presented as mean ± SD. Normality was verified with the Shapiro-Wilk test, and homogeneity of variances with the Levene’s test. Where these assumptions were verified, possible differences between the EXP and CON groups before and after the intervention were determined by the Student's *t*-test for independent data. Possible intra-group differences were tested using the *t*-test for paired data.

In cases where normality or homogeneity of variance was not verified, the Mann-Whitney U test was used to determine possible differences between groups. The Wilcoxon signed-rank test was applied to determine possible intra-group differences pre- and post-intervention.

When parametric statistics were employed, Cohen's *d* was used to determine the effect size, establishing values equal to or less than 0.20 as “no effect”, values between 0.21 and 0.49 as “small effect”, values between 0.50 and 0.79 as “moderate effect” and values equal to or greater than 0.80 as “large effect” (Caycho et al., 2016). In non-parametric statistics, the effect size was determined by Rank Biserial Correlation. Following Goss-Sampson (2019), the interpretation was similar to Pearson's r correlation statistic. Consequently, r_rb_ values of less than 0.10 were established as trivial effect sizes, between 0.10 and 0.29 as weak, between 0.30 and 0.49 as moderate, and greater than 0.50 as large.

In all cases, a significance level of *p* < 0.05 was established. Calculations were performed with the free statistical software JASP (Version 0.16.4; JASP Team, 2022).

## Results

Of the 30 players selected, six did not perform the second evaluation (two from the EXP and four from the CON group), thus they were not considered for the analysis of the results. The reasons were, in four cases, musculoskeletal injuries; in one case, not tolerating the sensation of claustrophobia caused by the gas analyzer mask during the first assessment; and in one case, the athlete claimed lack of time to perform the tests. For this reason, the results presented correspond to 24 players, 13 from the EXP and 11 from the CON group. The players' characteristics before the intervention are presented in Table 1.

**Table 1:** Pre-intervention characteristics of CON and EXP groups

	CON (n = 11)	EXP (n = 13)	*p*-value
Age (y)	21.3 ± 2.6	22.7 ± 3.8	0.340
Body height (cm)	162.3 ± 9.5	168.5 ±7.2	0.095
Body mass (kg)	64.4 ± 10.0	71.3 ± 11.8	0.145

**Abbreviations:** CON = control group; EXP = experimental group

### Use of the POWERBreathe Device

Ten of the 13 players in the EXP group completed the 75 planned IMT sessions without any omission. The three remaining players did not complete all sessions; in two cases, the reason given was “forgetfulness”, completing 74 and 66 sessions, respectively; in the remaining case, the athlete stopped the intervention during week six on the advice of her physiotherapist, when she determined “pain in the dorsal area associated with diaphragmatic contracture”. She then continued the sessions during weeks seven and eight with no other reported effects, completing 65 sessions.

The resistance achieved by players at the end of the intervention corresponded to level 4.2 on average (SD = 0.8), with level 0 being the minimum and level 10 the maximum possible; this is equivalent to a resistance of 131.6 ± 22.6 cmH_2_O. The minimum level reached was 2.75 (88.5 cmH_2_O), and the maximum level was 5.25 (157.0 cmH_2_O).

### Physiological Variables at Rest

Table 2 shows the results of the resting spirometry. Before the intervention, when comparing both groups, a significant difference in FVC (*p* = 0.033) and PIF (*p* = 0.004) in favor of the EXP group was found. There was no significant difference between groups in FEV, FEV1/FVC, and MEF50 (*p* ≥ 0.05).

**Table 2:** Pre and post intervention respiratory function variables.

		CON (n = 11)			EXP (n = 13)	
Variable	Pre	Post	ES	Pre	Post	ES
FVC (L)	4.2 ± 0.6	4.3 ± 0.6	0.209 (*d*)	4.9 ± 0.7	4.9 ± 0.9	0.086 (*d*)
FEV1 (L)	3.5 ± 0.4	3.4 ± 0.5	−0.050 (*d*)	3.9 ± 0.6	3.7 ± 0.6	−0.109 (*d*)
FEV1/FVC	0.83 ± 0.07	0.81 ± 0.07	−0.306 (*d*)	0.73 ± 0.23	0.77 ± 0.09	0.26 (*d*)
MEF50 (L/s)	3.9 ± 1.0	3.8 ± 0.9	−0.217 (*d*)	3.9 ± 1.2	4.0 ± 1.1	0.272 (*d*)
PIF (L/s)	4.7 ± 1.2	4.1 ± 1.4	−0.052 (*d*)	6.6 ± 1.5	6.3 ± 1.6^#^	−0.345 (*r_rb_*)

**Abbreviations:** CON = control group; EXP = experimental group; ES = effect size; FVC = Forced Vital Capacity; FEV1 = Forced Expiratory Volume in 1 s; MEF50 = maximal expiratory flow at 50% of the forced vital capacity; PIF = Peak Inspiratory Flow; d= Cohen's d; r_rb_ = Rank Biserial Correlation; ^#^= significant difference (p < 0.05) between groups post-intervention.

After the experimental protocol, the intra-group comparison post- vs. pre-intervention showed no significant differences between the first and the second evaluation (*p* ≥ 0.05) for any of the variables in any of the groups. In the post-intervention inter-group comparison, it was observed that for PIF, the difference continued to be significant in favor of the EXP group (*p* = 0.005), while for FVC no significant difference was observed between groups.

### Physiological Variables during Exercise

Table 3 shows the incremental progressive test results. All but two of the participants reached maximum RER values ≥ 1.10, a criterion commonly used to determine VO_2max_. One CON athlete did not reach this value in both tests, while one EXP athlete did not reach this value only in the post-intervention test. Nevertheless, both athletes exceeded the value of 1.0 in all cases, a criterion also used by some authors (Midgley et al., 2007).

**Table 3:** Ergospirometric variables in CON and EXP groups pre- vs. post-intervention

		CON (n = 11)			EXP (n = 13)	
Variables	Pre	Post	SE	Pre	Post	SE
Absolute VO_2max_ (L/min)	2.1 ± 0.2	2.2 ± 0.3*	0.820 (*d*)	2.3 ± 0.3	2.4 ± 0.2	0.293 (*d*)
Relative VO_2max_ (ml/kg∙min)	33.6 ± 3.6	34.5 ± 3.2*	0.881 (*d*)	34.0 ± 3.9	35.0 ± 4.4	0.334 (*d*)
VE_max_ (L/m)	86.5 ± 10.3	89.1 ± 11.0	0.281 (*d*)	93.2 ± 16.8	96.5 ± 17.5	0.188 (*d*)
RER_max_	1.2 ± 0.1	1.2 ± 0.1	0.279 (*d*)	1.2 ± 0.1	1.1 ± 0.05	−0.309 (*d*)
pVO_2max_ (W)	196.4 ± 24.6	188.2 ± 27.1^#^	−1.000 (*r_rb_*)	212.5 ± 20.1	212.3 ± 22.8^#^	0.000 (*r_rb_*)
VT1 absolute (L/min)	1.5 ± 0.2	1.4 ± 0.3	−0.288 (*d*)	1.6 ± 0.3	1.7 ± 0,2	0.514 (*r_rb_*)
VT1 relative (ml/kg∙min)	23.7 ± 2.9	21.9 ± 2.3	−0.394 (*d*)	22.8 ± 3.0	24.8 ± 4.7	0.273 (*r_rb_*)
VE (VT1) (L/m)	42.3 ± 6.0	40.8 ± 6.2	0.523 (*d*)	45.2 ± 9.6	46.8 ± 8.7	0.223 (*d*)
RER (VT1)	0.97 ± 0.07	0.95 ± 0.04	−0.468 (*d*)	0.96 ± 0.06	0.94 ± 0.05	−0.292 (*d*)
pVT1 (W)	102.6 ± 26.4	94.6 ± 26.0#	−0.433 (*d*)	117.0 ± 27.6	117.8 ± 16.6^#^	0.028 (*d*)
RPE at 120 W	4.2 ± 1.5	4.3 ± 1.0	0.257 (*d*)	4.2 ± 0.7	4.7 ± 0.9*	0.425 (*r_rb_*)

**Abbreviations:** CON = control group; EXP = experimental group; ES = effect size, comparing pre- vs. post-intervention; VEmax= pulmonary ventilation associated with VO2max; RERmax= respiratory exchange ratio associated with VO2max; pVO2max= power associated with VO2max; VT1 = first ventilatory threshold; VE (VT1) = pulmonary ventilation associated with VT1; RER (VT1) = respiratory exchange ratio associated with VT1; RPE = Rate of Perceived Exertion; d = Cohen's d; rrb = Rank Biserial Correlation; * = significant difference (p < 0.05) pre- vs. post-intervention; #= significant difference (p < 0.05) between groups post- intervention.

Pre-intervention, none of the variables analyzed showed a statistically significant difference. Concerning the intra-group comparison pre- vs. post-intervention, after the intervention, only the CON group showed a significant improvement (*p* < 0.05) in absolute and relative VO_2max_, with a large effect size for both variables (*d* ≥ 0.80). Additionally, post-intervention, a significant increase in the RPE was observed at the end of stage 4 in the EXP group; in the CON group, there was also an increase in this variable, but it was not significant (*p* ≥ 0.05).

For all the other variables analyzed, except for VE_max_, a decrease was observed in the CON group in the second evaluation compared to the first one. In the EXP group, on the contrary, all variables increased or maintained their values post- vs. pre-intervention. However, there were no significant intra-group differences between the two assessments.

Concerning the post-intervention inter-group comparison, a significant difference was determined in the variables of intensity associated with VO_2max_ (iVO_2max_) with a moderate effect size (*p* = 0.028; r_rb_ = 0.497) and intensity associated with VT1 (iVT1) with a large effect size (*p* = 0.016; *d* = 1.099) in favor of the EXP group. For the other variables, no significant post-intervention differences were observed between both groups.

### Subjective Perceptions Associated with the Use of Powerbreathe

Seven participants of the EXP group reported some discomfort associated with using POWERBreathe, while the remaining six players reported not having experienced any physical discomfort. Of the former, two athletes reported that while using POWERBreathe, their ears “stopped up”, which happened mainly during the first few weeks and then subsided. Two other participants reported pain or contracture in the cervical musculature, particularly during the last few weeks. One of these participants also reported feeling “a little dizzy” associated with this cervical pain. Two athletes reported nausea at the end of the 30 breaths, particularly during the last few weeks; in all cases, the nausea was mild and subsided quickly. Finally, as previously mentioned, one athlete reported “stitch-like pain in the dorsal area” that her physiotherapist linked to a possible “diaphragmatic contracture” that forced her to suspend IMT for a week.

Regarding the effort required by participants to perform IMT, the average value reported (on a scale from 0 to 10) was 5.7 ± 1.9, with a minimum of 2 and a maximum of 8. During the intervention, eight of the 13 players in the EXP group (62%) experienced a feeling of improvement, which they assumed was associated with using the device. The other five participants (38%), on the other hand, did not perceive any significant improvement. Considering the former, in all cases, they referred to such improvements in the “last two or three weeks” of intervention, describing the subjective perception using the expressions “I was less agitated”, “I had more air” or “I could play the whole game without choking”.

## Discussion and Conclusions

To our knowledge, this is the second study where the effect of IMT in handball was investigated (the first being Hartz et al., 2018) and the first in female handball players. Hartz et al. (2017) studied the effects of POWERBreathe in this population, but as part of a warm-up protocol and not as IMT *per se*. Given the anatomical and functional differences in the respiratory system between men and women (LoMauro and Aliverti, 2018) and the possible greater benefit of IMT in the latter (Riganas et al., 2019), we consider their study relevant in athletes of both sexes. Additionally, unlike the other papers reviewed, in our study, we followed the IMT protocol suggested by the manufacturers of this device. This aimed to determine the effects of its use under similar conditions to those that athletes would use in a real-life context.

In the present study, after eight weeks of IMT, no significant improvements were observed in spirometric variables at rest. This finding agrees with that reported by Najafi et al. (2019) in youth soccer players, who, after a similar period of IMT, found no improvements in the analyzed spirometric variables. In contrast, other authors observed improved spirometric variables at rest in endurance runners with an IMT protocol of similar duration to the present study (Rozek-Piechura et al., 2020). Regarding the variables measured during the progressive incremental test, a significant difference in favor of the EXP group was observed in power associated with VO_2max_ (pVO_2max_) and power associated with the VT1 threshold (pVT1) only. In the first case, the effect size was moderate (0.30 > r_rb_ < 0.49), while in the second case, the effect size was large (*d* > 0.80).

IMT used in our intervention focused on developing the strength of inspiratory musculature. In this sense, it would be expected that, as an adaptation, a lower demand of the inspiratory muscles for the same exercise load would be observed, with the consequent reduction of the percentage of cardiac output destined to the inspiratory musculature; thus confirming an increase in the flow of oxygenated blood available for the aerobic work of the peripheral musculature, directly involved in the execution of the technical movement (Gama et al., 2021). This physiological phenomenon could explain, at least in part, the increase mentioned above in pVT1 in the EXP compared to the CON group. These findings could, in turn, partially explain the improvement in aerobic performance reported by Hartz et al. (2018) after 12 weeks of IMT in male handball players.

In our study, no significant improvements associated with IMT were observed in absolute or relative VO_2max_, nor VO_2_ associated with VT1. These results indicate that, for this population and following the protocol indicated by the device manufacturers, eight weeks of IMT are not beneficial for increasing VO_2_ at maximal or submaximal efforts. This finding agrees with other studies, where no improvement in VO_2max_ was detected after four weeks of IMT in runners (Williams et al., 2002) or after six weeks of IMT in highly trained rowers (Riganas et al., 2008).

In the present work, this aspect could be partially explained by the high level of training of the athletes that composed the sample. Forty-two percent (10 out of 24) of them had recently participated in international competitions, representing the Uruguayan national team. This fact, and the fact that athletes were in the middle of the competitive period, suggests that the level of adaptation of physiological variables was close to their maximum potential, which would make it challenging to achieve additional improvements. This aspect could, in turn, help explain the absence of differences recorded in spirometric variables at rest. Nevertheless, it appears to be inconsistent with the relatively low VO_2max_ levels found in our sample (approximately 34 ml/kg∙min on average), which is lower than values close to 50 ml/kg∙min reported by other authors for elite-level female handball players (Michalsik and Aagaard, 2015). We believe this could be due to using a cycloergometer instead of a treadmill for measuring this variable, which may have limited the results.

In contrast to the above-mentioned, other studies did find improvements in at least some of these variables in high-level athletes. In this regard, Olivera et al. (2017) reported that four weeks of POWERBreathe use in professional male basketball players resulted in a significant increase in PIF (*p* < 0.05). It should be mentioned, nevertheless, that their study used a small sample of players (n = 7), and there was no comparison with a control group. In another study performed with elite male rugby players, the author reported that IMT for four weeks, four weekly sessions, and using the same device as the one used in the current study, resulted in a significant improvement in the players' lung capacity. Precisely, a significant improvement (*p* < 0.05) in FEV1, FVC, PFE, and the Tiffeneau index (FEV1/FVC) was confirmed only in the experimental group (Albert, 2020). For this reason, we are aware that the high level of physical fitness of players in our study represents only a partial explanation for the absence of improvements in these variables.

In relation to the RPE, an increase was verified at the end of stage 4, with no significant differences (*p* ≥ 0.05) between the two groups post-intervention. Contrary to what might be expected, IMT did not lead to a decrease in the RPE at specific submaximal intensities in the EXP group. This finding is in contrast to what was reported by Lorca-Santiago et al. (2020), who suggested that IMT would be effective in reducing the sensation of fatigue and dyspnea in intermittent sports, as is the case of handball. It is noteworthy that, of all the athletes studied, only one participant had previously experienced the Borg scale, while the rest of players reported never having used it before. Given the subjective nature of this tool and the need for previous experience in its use, we consider that these results should be interpreted with caution.

Additionally, regarding the subjective aspects of using POWERBreathe, we observed considerable variability in the effort required for its use and in the eventual benefits perceived by participants after its use. As previously noted, on average, athletes perceived the effort required to its use as moderate (5.7 out of a maximum of 10), and none of them mentioned that they would not use it again. Regarding the subjective perception of improved performance resulting from using the device, a non-negligible percentage of participants (8 out of 13, 62%) mentioned a perception of improved aerobic capacity associated with IMT. This could be due to a placebo effect (Bérdi et al., 2011), other psychobiological aspects that influence the perception of effort (Van Cutsem et al., 2017), or other variables associated with performance not addressed in our study. Most athletes mentioned that such improvement was perceived after three to four weeks of intervention. It is worth noting that the product manual indicates that the users perceive the benefits of IMT after approximately four weeks of training, which would agree with what was found in this work.

Some particularities of the present study could be perceived as limitations and eventually be considered in future research. In this sense, although athletes performed the evaluations outside the menstrual period (so they were not in the first days of the follicular phase), the precise stage of their cycle was not known at the time of evaluation. However, previous works did not find differences in variables concerning aerobic capacity (Oğul et al., 2021) or perceived exertion (Bonetti et al., 2021) as a function of the stage of the menstrual cycle.

We also recognize that, considering the characteristics of handball, it would have been preferable to use a treadmill (instead of a cycloergometer) to conduct the progressive incremental tests. Although the initial intention had been to use a treadmill, it was not available at the time of the study. As for the progressive exercise protocol applied, its selection was due to the fact that the stabilization of physiological variables that tends to occur at each stage facilitates the recording of the RPE (Zuniga et al., 2012). However, we admit that, for the determination of ventilatory thresholds, a ramped protocol would have been more appropriate (Michalik et al., 2019).

An additional limitation was that, due to restrictions imposed by the pandemic caused by COVID-19, the usual technical-tactical training sessions were altered during the last two weeks of intervention. This aspect could have influenced the minor improvements observed in the variables analyzed. The possible detraining associated with the decrease in exercise volume during this period could partly explain some unexpected findings, such as the reduction (although not statistically significant) in PIF values post- vs. pre-intervention. Nevertheless, we emphasize that these modifications were identical for both groups and that all the participants in the EXP group continued with IMT as planned.

In summary, these findings suggest that, for the population studied, (i) the use of POWERBreathe is feasible in terms of time, effort, and side effects (few and not relevant), (ii) at least three to four weeks of its use would be necessary for athletes to detect possible improvements, and (iii) if benefits are confirmed, they would be scarcely significant.

We believe that the results obtained represent a novel contribution, as this is the first study to analyze the physiological effects of IMT in women's handball. We encourage further studies in this population that implement biochemical and other complementary analyses and incorporate more specific assessments for this sport. We also consider it pertinent to conduct similar studies in other sports populations, such as boxing. In boxing, the use of IMT has been little studied despite its possible benefits for improving performance, given the characteristics of this sport.
